# Hospitals and the Law of Nuisance

**Published:** 1910-03-05

**Authors:** 


### SPECIAL ARTICLE.

### HOSPITALS AND THE LAW OF NUISANCE.

The law of nuisance is sometimes a matter of considerable interest to hospital authorities. How far can their building and other operations be interfered with by those who allege that to be near a hospital is to be near a "nuisance" in the legal sense of the term?

The law—which is to be gathered from cases, not statutes—has fluctuated widely during the last century and a half, and now stands very much where it did before that time. According to a case decided in 1752,[1] it was proposed by certain persons to erect a building in Cold Bath Fields to be used as a hospital for the reception of small-pox patients. The plaintiff, who was owner of building land in the neighbourhood, sought an injunction to restrain the building on the ground that fears of infection from the proposed hospital would deteriorate his letting values. Lord Hardwicke, in refusing the injunction, said that loss arising from the fears of mankind, though reasonable, would not create a nuisance at law, and that before he could grant an injunction he must be satisfied that what was proposed to be done would be a legal nuisance affecting the plaintiff's private rights. "I think," said he, "such a charity is likely to prove of great advantage to mankind, and such a hospital must not be far from a town." Since the days of Lord Hardwicke, however, the courts have until quite recently encouraged those who would prefer these claims against hospitals.

The leading case on the subject was heard in 1881.[2] There damages were claimed against the Metropolitan Asylums Board owing to the use of their hospital at Hampstead for small-pox and other infectious diseases; and an injunction was asked for to restrain them. The decision of the case chiefly turned on the plea set up by the defendants, namely, that as they were acting under statutory powers and performing a statutory duty they could not be held liable. It was held that this defence could not prevail. Lord Blackburn, in giving judgment in the House of Lords, said: "I think that to gather together in one spot patients suffering from infectious disease is lawful, but it must be made under such guards as not to endanger the public health by communicating this infectious disease, and, as it seems to me, as not to produce injury to the rights of the owners of adjoining property by producing a nuisance to it." The jury in that case actually found that there was a nuisance. A few years later an injunction[3] was granted by Mr. Justice Stirling, acting on the report of a medical referee to the effect that there was an appreciable danger of infection by aerial convection to the property of the plaintiff.

Since 1888 there have been many cases heard of alleged nuisance or threatened nuisance, but they have as a general rule been dismissed. There has been a kind of reverter to the tolerant spirit advocated by Lord Hardwicke, whose enlightened principles were recently adopted and approved by Mr. Justice Farwell.

In 1904 an action[4] was brought against the Nottingham Corporation to prevent their using a certain building as a small-pox hospital, on the ground that it was likely to be both a public and a private nuisance. The judge held that as no actual nuisance had arisen, and the action was *quia timet*, the plaintiffs were bound to show a strong probability that the apprehended mischief would in fact arise. He also found, as a result of the evidence, that the site of the hospital was carefully chosen and constituted no appreciable danger to the public health and no nuisance to the plaintiff's property. The hospital in question was fifty-one feet from the high road, within a quarter of a mile of 204 residents, and half a mile of 510 residents, and also near a colliery in which 1,280 men worked. Mr. Justice Farwell said: "The theory of aerial convection or dissemination of the disease of small-pox has not received the unequivocal sanction of medical science, and the establishment of a small-pox hospital properly conducted is not of itself necessarily such a serious source of danger to persons resident, working, or passing by in its immediate vicinity—say, a radius of 50 feet—as to constitute a public or a private nuisance for which an injunction will lie in a *quia timet* action."

The same principle was adopted in Ireland in the same year,[5] when Lord Justice Fitzgibbon said: "It seems probable that the dread of small-pox is to a great extent the result of tradition. That scourge of the eighteenth century retains its terrors for those who do not realise that it has been deprived of most of its dangers. . . . An isolation hospital reduces the risk for every inhabitant of the district. It is in fact a necessity, and though the individual must be protected, the public advantage should not be forbidden unless the danger and injury to the individual are clearly proved."

Mr. Murray draws the following inference from the decided cases[6]: "The general conclusion from the authorities is that in cases of apprehended nuisance the court is not ready to interfere with an infectious hospital unless a strong case of real danger is made out; that while medical science discovers fresh theories of infection, so are new means of prevention invented, and that mere personal inconvenience or anxiety must yield to the health and welfare of the general community."

Apart from cases in which claims for apprehended nuisance of a general nature are made against hospitals, and in which, as we have seen, the chances of the plaintiff's success are not very good, there may be cases in which the hospital will be held liable. For instance, where a local authority used a stable near the plaintiff's house as a small-pox hospital, with the result that the plaintiff's daughter fell ill and died of the disease, the local authority had to pay damages.[7]

Again, if hospital authorities were to negligently allow an infectious patient to go forth and spread infection, they might be held liable unless (as in one case actually happened[8]) they acted on the advice of their visiting physician, for whose actions they could not be held responsible.

Great care should be taken by those who have the management of isolation hospitals to prevent trespassers from encroaching. An adult trespasses at his own risk, but with children the law may be very different. For instance, in Sherwell *v.* Alton Urban District Council (1909), 25 T.L.R., 417, it appeared that an isolation hospital had been erected owing to an outbreak of diphtheria on land adjoining the house in which the plaintiff lived with his family. One of the plaintiff's children, aged seven, caught diphtheria. Four days previously she had crawled under the wire fence separating the hospital from the plaintiff's land and had approached the hospital where the infected children and nurses were. She had also played with a kitten which had lived at the hospital. It was held that there was no evidence of negligence on the part of the defendants to go to the jury, as the child might have caught the disease in many different ways.

To the non-legal mind it may seem incredible that such a claim was preferred at all; but if it could have been shown that the child became infected owing to her visit to the hospital, the authorities of that institution might have been held liable. True the child was a trespasser, but the law is very lenient to children. If a person leaves open, or allows a child to stray through, a gap in his hedge, he may be held responsible if a child, following the bent of its childish nature, trespasses and gets into mischief. For instance, in a case decided last year some children trespassed through a gap in a hedge on to the property of a railway and began playing with a disused turntable. One of the children was injured, and an action for negligence was brought against the railway company. It was held (by the House of Lords) that although the defective condition of the hedge was not the effective cause of the accident, yet that there was evidence on the part of the railway company to go to the jury.

---

[1] Baines *v.* Baker, Amb. 159. [2] Metropolitan Asylums District *v.* Hill, 1881, 4 Q.B.D. 433. [3] Bendelow *v.* Wortley Union, 1888, 57 L.J.Ch. 762. [4] A. G. *v.* Nottingham Corporation (1904), 1 Ch. 673. [5] Attorney-General *v.* Rathmines and Pembroke Joint Hospital Board (1904), 1 Ir. R. 161. [6] The Law of Hospitals, p. 199. [7] Chapman *v.* Gillingham U.D.C., *Times*, March 28, 1903. [8] Evans *v.* Liverpool Corporation, 1906, 1 K.B. 160.

